# Lineage rewiring in lung adenocarcinoma via HNF4α and NKX2-1 dynamics

**DOI:** 10.1101/gad.353142.125

**Published:** 2025-09-01

**Authors:** Alice Feng, Alena Yermalovich, Matthew Meyerson

**Affiliations:** 1 Department of Medical Oncology, Dana-Farber Cancer Institute, Boston, Massachusetts 02215, USA;; 2 Harvard College, Cambridge, Massachusetts 02138, USA;; 3 Broad Institute of Harvard and Massachusetts Institute of Technology, Cambridge, Massachusetts 02142, USA;; 4 Center for Cancer Genomics, Dana-Farber Cancer Institute, Boston, Massachusetts 02215, USA;; 5 Departments of Genetics and Medicine, Harvard Medical School, Boston, Massachusetts 02115, USA

**Keywords:** KRAS inhibition, hybrid identity, lineage plasticity, lineage specifiers, lung adenocarcinoma

## Abstract

Lineage plasticity drives treatment resistance in lung adenocarcinoma (LUAD) as cancer cells adopt new identities. In this issue of *Genes & Development*, Fort and colleagues (doi:10.1101/gad.352742.125) report HNF4α as a key regulator of hybrid identity states and tumor progression in NKX2-1-positive LUAD. Using murine and human models, they show that HNF4α promotes gastrointestinal/liver-like programs and suppresses pulmonary identity by modulating cell identity-specific binding of NKX2-1. In addition, RAS/MEK signaling was implicated in maintenance of this hybrid identity state by regulating NKX2-1 chromatin binding in LUAD. These findings nominate HNF4α as a driver of LUAD plasticity and a potential therapeutic target to overcome resistance.

## Abstract (precis)

In this Outlook, Feng et al. discuss a study in this issue of *Genes & Development* by Fort et al. that shows that HNF4α and NKX2-1 work together to determine hybrid lineage identity states—and subsequently tumorigenesis—in lung adenocarcinoma (LUAD). Feng et al. posit that understanding the molecular, mechanistic link between HNF4α and NKX2-1 could lead to new avenues—targeting lineage plasticity—for developing combinatorial therapies and diagnostic strategies in LUAD and other cancers.

Lung cancer is the leading cause of cancer mortality, leading to >120,000 deaths per year in the United States (Siegel et al. 2025) and 1.8 million deaths annually worldwide (Zhou et al. 2024). The most common subtype of lung cancer is lung adenocarcinoma (LUAD), which most commonly arises from a subset of alveolar type 2 (AT2) cells. Despite significant advancements in chemotherapy, immunotherapy, and targeted therapy for LUAD, these treatments often suffer from resistance. An important cause of treatment resistance is lineage plasticity, which is when cancer cells exhibit a variety of cellular identities and differentiation states, resulting in loss of dependency on oncogenic drivers that are treatment targets (Quintanal-Villalonga et al. 2020). Lineage plasticity can also be observed in association with clinical resistance to targeted therapies. For example, targeted therapies have been observed to be followed by the conversion of LUAD lung cells to a neuroendocrine lineage, with *TP53*, *RB1*, and *EZH2* alterations identified as being associated with this conversion (Shi et al. 2023). Understanding the molecular drivers of lineage plasticity in LUAD and the various cellular identities that LUAD exhibits is therefore crucial in combatting treatment resistance and improving treatment efficacy.

Transcription factor NKX2-1, which is essential for regulating pulmonary differentiation, is expressed in 75%–85% of LUAD cases. The role of NKX2-1 in LUAD is complex, as on one hand, it is the major target of focal amplification in LUAD (Weir et al. 2007); on the other hand, downregulation of NKX2-1 leads to altered cell differentiation and a worse prognosis (Snyder et al. 2013), and there are *NKX2-1* loss-of-function mutations in some LUADs, notably in the mucinous subtype (Hwang et al. 2016). In a recent study, Pulice and Meyerson (2025) reported that NKX2-1 drives an alveolar differentiated state by controlling its enhancer's accessibility in a dosage-dependent manner and that the most significant focal amplification in LUAD is the amplification of the *NKX2-1* superenhancer. Furthermore, Pulice and Meyerson (2025) showed that *NKX2-1* confers persistence to EGFR-targeted therapies, aligning with EGFR's potential role in LUAD lineage plasticity.

In previous work, using a murine model, Snyder et al. (2013) demonstrated that Nkx2-1 loss leads to cellular identity changing from pulmonary to gastric lineage, and this mechanism involves transcription factors Foxa1 and Foxa2 (Foxa1/2) binding to gastric targets instead of pulmonary targets in the absence of Nkx2-1. In human NKX2-1-positive LUAD, FoxA1/2 also drive hybrid identity differentiation, an intermediate state of LUAD lineage plasticity when there is coexpression of pulmonary and gastrointestinal transcripts. Specifically, there is coexpression of NKX2-1 and HNF4α, a master regulator for gastrointestinal and liver cell identity (Orstad et al. 2022). Hence, understanding HNF4α’s role in NKX2-1-positive LUAD and its molecular relationship with NKX2-1 could greatly help characterize lineage plasticity and tumor progression in LUAD.

In a new study, Fort et al. (2025) identified HNF4α as a key driver of tumor growth and hybrid identity states in NKX2-1-positive LUAD by integrating murine and human models along with pancancer LUAD data from The Cancer Genome Atlas (TCGA) database. Analysis of TCGA LUAD patient data revealed that high *HNF4*α expression correlates with worse overall survival in patients with *NKX2-1*-high tumors. Consistently, conditional deletion of *Hnf4a* in genetically engineered mouse models (GEMMs) with Kras-driven, *Trp53*-deleted LUAD tumors resulted in significantly reduced tumor growth and decreased tumor cell proliferation, highlighting the importance of Hnf4a for LUAD growth in vivo. The investigators derived dual-positive organoids from the *Kras*, *Trp53*, and *Hnf4a* mutated (KPH) GEMMs, meaning that Nkx2-1 and Hnf4a were coexpressed. Hnf4a inhibition in dual-positive organoids significantly increased cell death and impaired proliferation, whereas exogenous HNF4α expression in dual-positive human LUAD enhanced in vivo growth.

Next, Fort et al. (2025) found that HNF4α activates gastrointestinal/liver-like differentiation programs in NKX2-1-positive LUAD models, directly binding to and activating targets like *LGALS4*, *HNF1A*, and *MUC13* in both mouse and human cell experiments. At the same time, HNF4α suppresses pulmonary identity programs in hybrid identity LUAD, including AT2 and AT1 cell markers like *Sftpc*, *Ager*, and *Cav1* in mouse models and NKX2-1 targets including *LMO3* and *SPOCK1* in human lung cancer cells. This was an unexpected finding because HNF4α is not typically expressed in the lung or in lung precursor cells. Aiming to determine the molecular relationship between HNF4α and NKX2-1, Fort et al. (2025) used complementary experimental techniques to show that HNF4α, NKX2-1, and FoxA1 are in close proximity and may form a novel protein complex. Interaction with HNF4α appears to redirect NKX2-1 binding from specific for pulmonary gene loci to encompassing both pulmonary and gastrointestinal gene loci. This shift appears to disrupt NKX2-1's ability to maintain a pulmonary identity. NKX2-1 and HNF4α were also observed to co-occupy both gastrointestinal and pulmonary gene regulatory regions in hybrid identity LUAD, a divergence from their mutually exclusive binding patterns in normal tissue.

Finally, Fort et al. (2025) investigated the RAS/MEK signaling pathway because recent studies suggest that its activation is associated with poorly differentiated LUAD and with HNF4α expression. They found that inhibition of RAS/MEK signaling reduced gastrointestinal/liver gene expression and enhanced AT1 and AT2 alveolar signatures. MEK inhibition also led to widespread redistribution of NKX2-1 binding, increasing association with alveolar gene loci and reducing association with gastrointestinal gene loci. This resembles the effects of *Hnf4*α deletion and suggests that RAS/MEK signaling may modulate lineage plasticity in alveolar cells by regulating NKX2-1 chromatin binding.

In conclusion, Fort et al. (2025) show that coexpression and interaction between HNF4α and NKX2-1, which define gastrointestinal and alveolar lineages, respectively, result in a rewiring of cellular identity, promoting cell proliferation and tumor growth. Additionally, active RAS/MEK signaling can sustain these hybrid identity states. These findings open the doors for the consideration of new strategies in LUAD treatment, including envisioning the targeting HNF4α and its upstream regulators, like FoxA1/2, in combination with targeted inhibitors. HNF4α could also be evaluated as a biomarker for treatment prognosis and resistance in LUAD. The concept of hybrid identity states and the discovery of the NKX2-1–HNF4α complex could also extend beyond LUAD and inform treatment strategies for other cancers affected by lineage plasticity.
